# Discovery of Spatial Peptide Signatures for Neuroblastoma Risk Assessment by MALDI Mass Spectrometry Imaging

**DOI:** 10.3390/cancers13133184

**Published:** 2021-06-25

**Authors:** Zhiyang Wu, Patrick Hundsdoerfer, Johannes H. Schulte, Kathy Astrahantseff, Senguel Boral, Karin Schmelz, Angelika Eggert, Oliver Klein

**Affiliations:** 1 BIH Center for Regenerative Therapies BCRT, Charité—Universitätsmedizin Berlin, 13353 Berlin, Germany; zhiyang.wu@charite.de; 2 Department of Pediatric Oncology, Helios Klinikum Berlin-Buch, 13125 Berlin, Germany; patrick.hundsdoerfer@helios-gesundheit.de; 3 Department of Pediatric Oncology & Hematology, Charité—Universitätsmedizin Berlin, 13353 Berlin, Germany; johannes.schulte@charite.de (J.H.S.); kathy.astrahantseff@charite.de (K.A.); karin.schmelz@charite.de (K.S.); angelika.eggert@charite.de (A.E.); 4 Partner Site Berlin, The German Cancer Consortium (DKTK), 10117 Berlin, Germany; 5 The German Cancer Research Center (DKFZ), 69120 Heidelberg, Germany; 6 Institute of Pathology, Charité—Universitätsmedizin Berlin, 10117 Berlin, Germany; senguel.boral@charite.de; 7 Berlin Institute of Health, Charité—Universitätsmedizin Berlin (BIH), 10178 Berlin, Germany

**Keywords:** neuroblastoma, risk assessment, intratumor heterogeneity, peptide signatures, MALDI-MSI

## Abstract

**Simple Summary:**

The childhood tumor, neuroblastoma, has a broad clinical presentation. Risk assessment at diagnosis is particularly difficult in molecularly heterogeneous high-risk cases. Here we investigate the potential of imaging mass spectrometry to directly detect intratumor heterogeneity on the protein level in tissue sections. We show that this approach can produce discriminatory peptide signatures separating high- from low- and intermediate-risk tumors, identify 8 proteins aassociated with these signatures and validate two marker proteins using tissue immunostaining that have promise for further basic and translational research in neuroblastoma. We provide proof-of-concept that mass spectrometry-based technology could assist early risk assessment in neuroblastoma and provide insights into peptide signature-based detection of intratumor heterogeneity.

**Abstract:**

Risk classification plays a crucial role in clinical management and therapy decisions in children with neuroblastoma. Risk assessment is currently based on patient criteria and molecular factors in single tumor biopsies at diagnosis. Growing evidence of extensive neuroblastoma intratumor heterogeneity drives the need for novel diagnostics to assess molecular profiles more comprehensively in spatial resolution to better predict risk for tumor progression and therapy resistance. We present a pilot study investigating the feasibility and potential of matrix-assisted laser desorption/ionization mass spectrometry imaging (MALDI-MSI) to identify spatial peptide heterogeneity in neuroblastoma tissues of divergent current risk classification: high versus low/intermediate risk. Univariate (receiver operating characteristic analysis) and multivariate (segmentation, principal component analysis) statistical strategies identified spatially discriminative risk-associated MALDI-based peptide signatures. The AHNAK nucleoprotein and collapsin response mediator protein 1 (CRMP1) were identified as proteins associated with these peptide signatures, and their differential expression in the neuroblastomas of divergent risk was immunohistochemically validated. This proof-of-concept study demonstrates that MALDI-MSI combined with univariate and multivariate analysis strategies can identify spatially discriminative risk-associated peptide signatures in neuroblastoma tissues. These results suggest a promising new analytical strategy improving risk classification and providing new biological insights into neuroblastoma intratumor heterogeneity.

## 1. Introduction

Neuroblastoma is a pediatric cancer arising in approximately 1 of 100,000 children under 15 years of age in Germany [1]. It is the most common malignant solid tumor diagnosed in infants with a median age at diagnosis of 17 months [2]. The tumor derives from neural crest cells of sympathoadrenal lineage, and can develop anywhere in the sympathetic nervous system. About 65% of primary tumors arise in the adrenal medulla or lumbar sympathetic ganglia, while the rest arise in the neck, chest and pelvis. Clinical behavior and outcome is highly diverse, ranging from low-risk disease with the highest rate of spontaneous regression in all cancers to treatment-refractory lethal disease progression or treatment-resistant relapse occurring in high-risk disease despite aggressive multimodal treatment [3,4]. Consequentially, neuroblastoma treatment recommendations range from mere observation or surgical resection alone to very aggressive therapy protocols including high-dose chemotherapy, irradiation and immunotherapy [5].To address the issue of appropriate therapy intensity, a common international staging and risk classification system (INSS/INRG) has been developed [6,7]. In Europe, patients have been classified into three risk groups following the criteria described in Table 1 [5].The additional International Neuroblastoma Pathology Classification (INPC) criterion is exclusively used in the USA [8]. Neuroblastoma samples from patients with low and intermediate risk (INSS/INRG) were grouped together for this retrospective study and high-risk patients were defined as in Table 1 (Stage 4 > 18 months plus all *MYCN*-amplified cases).

*MYCN* amplification was the first identified clinically relevant molecular biomarker for neuroblastoma [9], and remains a strong single predictor for unfavorable outcome. However, a recent report from the INRG revealed that the prognostic impact of *MYCN* amplification is greatly dependent on the context of clinical and biological features [10]. In Germany, current risk stratification for the ongoing clinical trials is based on patient age, stage, *MYCN* amplification and the result of an mRNA-based molecular classifier [11] that is continuously further improved on transcriptomic and genomic levels [12]. We previously demonstrated spatial intratumor genetic heterogeneity and first evidence of branched evolution in neuroblastoma by bulk sequencing of paired diagnostic and relapse tumor samples [13]. A more recent sequencing effort has demonstrated extensive genetic intratumour heterogeneity in neuroblastoma with distinct evolutionary patterns that impact clinical behavior [14]. Previous extensive next-generation sequencing efforts by the global neuroblastoma community to catalog genetic aberrations in neuroblastoma [12,14,15,16,17,18,19,20,21] used primarily single diagnostic biopsies, and identified a low number of recurrent point mutations and translocations even in high-risk and relapsed neuroblastomas. One of these studies also included matched diagnostic and relapse samples from five patients, corroborating evidence of the genetic evolution of disease [20]. The demonstration of intratumor genetic heterogeneity and its evolution over disease course have assisted an expansion of tissue sample collection accompanying patient treatment and trials worldwide. In-depth analysis and further interpretation with respect to potential clinical implications will achieve a better grasp of the extent of intratumor heterogeneity in neuroblastoma to improve personalized patient treatment. Knowledge remains limited about the influence of both high intratumor heterogeneity and peptide signatures in neuroblastomas on disease progression and response to treatment. Tumor progression, in general, is known to be affected by tumor cellular interplay and the surrounding microenvironment [22]. Taken together, there is unmet need for reliable neuroblastoma risk classification that takes the tumor microenvironment and spatial heterogeneity into account.

Matrix-assisted laser desorption/ionization mass spectrometry imaging (MALDI-MSI) innovative technology combines the comprehensive mass spectrometric technique with a conventional histological evaluation. It allows unsupervised (unlabelled) analysis of molecules (e.g., metabolites, proteins, peptide, lipids and glycans) directly on a single tissue section, preserving their spatial coordinates and generating a molecular intensity map displaying the spatial relative molecule abundance [23,24,25,26]. MALDI-MSI has several advantages over other techniques, such as nano-desorption electrospray ionization (DESI), secondary ion mass spectrometry (SIMS) and liquid extraction surface analysis. MALDI-MSI requires less time to preform measurements, and provides better spatial resolution for a larger mass range, which are all important prerequisites for potential clinical application. The mass range of DESI and SIMS are limited to 2000 Da and 1000 Da, respectively. Spatial resolution of DESI and LESA are much lower than MALDI-MSI. In the present study, tryptic peptides ranging from 600 to 3500 Da were analyzed with 50 µm resolution, which could not have been achieved by the other techniques [27]. Direct identification of proteins, from which the peptides (acquired by MALDI-MSI) stem, remains limited to only a few abundant proteins. Several studies have recently demonstrated that high-resolution MSI data combined with microproteomics (high-resolution mass spectrometry) from microdissected tissue sections enables retention of an aspect of spatial specificity and accurate protein assignment (high mass accuracy) [28,29,30]. This is a promising strategy to explore potential disease-relevant protein markers in small patient collectives, but is not well suited for large-scale studies because of the longer processing time both for microdissection and mass spectrometry and the higher cost. In contrast, spatially distinct signatures of peptide spectra, such as those extracted from MALDI tissue imaging data, can be obtained in high-throughput in a clinically feasible time frame at a lower cost, and could provide a new dimension to the current classification of distinct patient subgroups, and potentially assist prediction of disease progression and/or resistance development [31,32,33,34]. Therefore, MALDI imaging is a promising technology to aid histopathology tissue assessment in routinely used large-scale formats. MALDI-MSI has been used to classify tumor types [35], to predict a therapeutic strategy [36,37] and to act as a biomarker for indicating response to treatment [38,39]. This technology can interpret molecular tumor composition while preserving spatial morphology, providing important insights into tumor heterogeneity and its impact on tumor biology.

In this pilot study, we investigated the feasibility and potential of MALDI-MSI combined with uni- and multivariate statistical strategies to (1) determine discriminative peptide signatures for neuroblastomas designated as high or lower risk groups as a starting point for subsequent more fine-tuned comparisons in the same patient subgroup and (2) to explore neuroblastoma intratumor heterogeneity for the first time on the protein level. Our aim was to reach an initial proof-of-concept that peptide signatures are capable of adding a new useful dimension of novel information to current clinical and transcriptomic risk classification schemes for neuroblastoma.

## 2. Results

### 2.1. Discriminative Peptide Signatures Can Be Derived from MALDI-MSI Data to Identify Different Tumor Features

**Figure 1:**
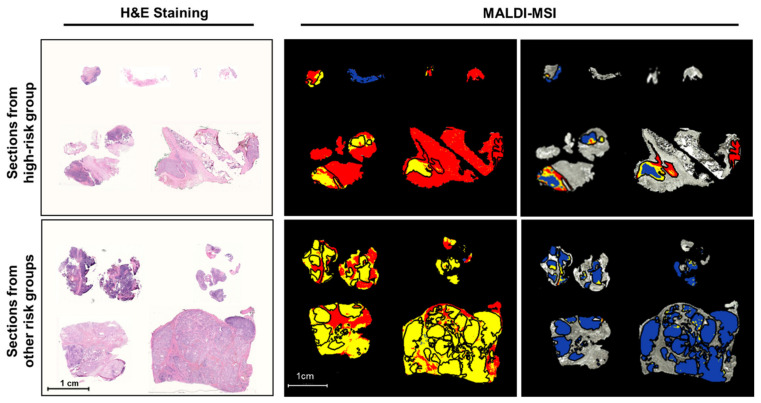
MALDI imaging identifies high-risk neuroblastomas by heterogeneous peptide signatures in tumor cell-rich regions. Sections from primary neuroblastomas with high or other risk classifications are shown with hematoxylin and eosin (H&E) staining for tissue section orientation in segmentation maps of MALDI-MSI analysis. Segments (indicated by different colors) represent different proteomic clusters generated by bisecting k-means clustering. Black lines surround tumor areas with >80% tumor cell content (annotated by the reference pathologist). Signatures derived from segmentation clustering across the whole tissue section are shown in the middle column and peptide signatures derived only across the tumor cell-rich areas in the sections shown on the right. Colors represent the same proteomic clusters in the 2 images in the middle column and the 2 images in the right column, but not between the middle and right images.

Here we evaluated the technical feasibility of MALDI-MSI to identify potential discriminative protein features of more aggressive neuroblastomas (high-risk) from formalin-fixed, paraffin-embedded (FFPE) tissue sections. Tissue samples were diagnostic biopsies from primary neuroblastomas categorized as high (*n* = 5) or other risk groups (low or intermediate risk, *n* = 4). Peptide signatures extracted from the analyzed tissue samples yielded 501 aligned m/z values in a mass range for tryptic peptides (m/z value range: 800–3200). Neuroblastoma cell-rich tumor regions yielded 397 aligned m/z values (Table S1). Representative average spectra of whole sections are shown in Figure S1. Peptide signatures were identified that characterized different tissue regions using bisecting k-means clustering, an unsupervised multivariate segmentation analysis, conducted on MALDI-MSI data from the tissue sections. Segmentation analysis produced two clusters shown as segmentation maps (Figure 1) that corresponded well to tissue areas in the tumors that were either tumor cell rich (>80%) or poor (defined by the reference pathologist). Consequently, peptide signatures obtained from MALDI-MSI data can distinguish tumor regions with a high tumor cell content from those with <80% tumor cell content directly from fixed tissue sections. To determine whether signatures could be defined to discriminate high from other risk groups, we performed a segmentation analysis (bisecting k-means) across only the regions with >80% tumor cell content, as defined by the pathologist. Unsupervised segmentation analysis of m/z values from these areas produced three segment clusters with different peptide signatures in high-risk tumors, (percentage of each peptide signature contributing to the tumor cell-rich region in Table S2), but only a single segment cluster in neuroblastomas were classified as lower risk (Figure 1). These data illustrate molecular intratumor heterogeneity for the first time on the protein level in high-risk tumors. Peptide signatures can be extracted from MALDI-MSI data by unsupervised clustering that correctly identify tumor cell-rich regions in neuroblastomas and discriminate high-risk neuroblastomas from lower risk groups.

**Figure 2:**
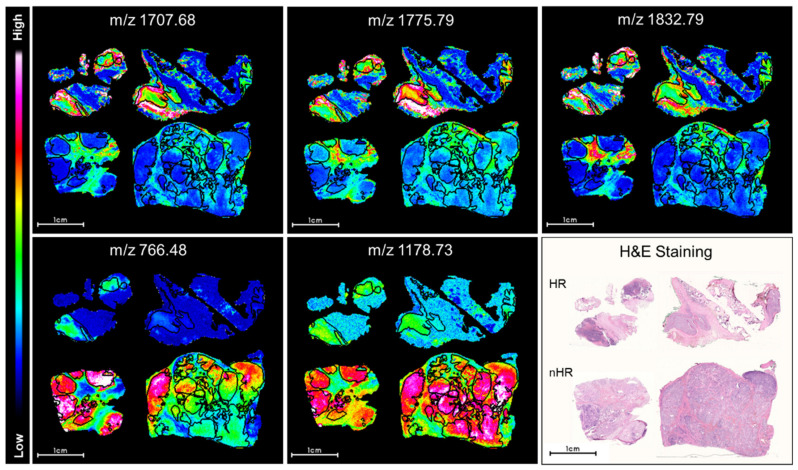
Selected peptides have differential intensity distributions in neuroblastoma cell-rich tumor regions between high and other risk groups. Relative peptide expression (color bar) is shown for MALDI m/z ion peaks with the highest significant area under the curve (AUC) values (>0.85, *p* < 0.001, top row) in receiver operator characteristic (ROC) analysis and the lowest AUC values (AUC < 0.3, *p* < 0.001, bottom MALDI images). MALDI-MSI ion images are shown for the same set of neuroblastoma tissue sections categorized either as high (HR) or other risk groups (nHR) in each image. Black lines surround tumor areas with >80% tumor cell content (annotated by the reference pathologist). Hematoxylin and eosin (H&E) staining in sections is shown for orientation.

Univariate analysis of MALDI-MSI data has the potential to determine which single peptides are the most discriminative between neuroblastoma tissues from different risk groups. We applied receiver operator characteristic (ROC) analysis to the total 397 aligned m/z peaks from tumor cell-rich areas in paired comparisons of tissue sections from high or other neuroblastoma risk groups. Differential spatial peptide intensity distributions in tissue samples from the two risk groupings determined the discriminatory power of individual peptides. Wilcoxon rank sum testing was applied to the total 397 aligned m/z peaks, resulting in 206 statistically significant m/z values (AUC values of >0.8 or <0.2; *p* < 0.001). From these, we show the five peptides with the strongest discriminatory values in Figure 2. Three peptides (m/z values: 1707.68, 1775.79 and 1832.79 Da) had significantly higher intensity distributions and two peptides (m/z values: 766.48 and 1178.73 Da) had significantly lower intensity distributions in tumor cell-rich regions from high-risk neuroblastoma tissue sections. To explore the potential of the most discriminatory peptides in the peptide signatures to discriminate high from other risk groups, principle component analysis was applied to the 206 statistically significant m/z values (AUC values of >0.8 or <0.2; *p* < 0.001). Principal component 1 (PC-1) mainly captured the differences within the tumor cell-rich regions in tumors from different risk groups and shows an increased intensity distribution in cell-rich tumor regions in high-risk neuroblastomas (Figure S2). Since 62% of the variance was explained by the first principal component (Figure S2), these findings demonstrate that both unsupervised and supervised statistical approaches result in discriminatory peptide signatures for high or other risk designations using MALDI-MSI data from neuroblastoma tissue sections.

### 2.2. Discriminative Proteins Were Identified from Neuroblastoma Tissue Sections Based on MALDI-MSI Data

To identify the proteins corresponding to the discriminatory tryptic peptide fragments, we used a bottom-up nanoliquid chromatography-tandem mass spectrometry (nanoLC-MS/MS) approach in adjacent tissue sections. This analysis assigned 147 of the 206 m/z values (Table S3) shown to be discriminative in ROC analysis (AUC > 0.7 or AUC < 0.3, *p* < 0.001) to peptides corresponding to proteins identified by nanoLC-MS/MS. According to guidelines, corresponding proteins to m/z values are correctly identified when the validating approach (nanoLC-MS/MS in this case; Table S4) identifies at least two peptides (detected in MALDI-MSI) from the same protein, whose spatial differential intensities are similar and correlated in the same tissue region (correlation coefficients) [40]. These guidelines were fulfilled for 8 proteins (Table 2) that corresponded to 18 MALDI-MSI m/z values. Of these 8 proteins, differential intensity distributions for m/z values from 6 (14 m/z values) proteins were verified using MALDI-MSI data obtained from 10 arrayed cores from neuroblastoma tissue areas having >80% tumor cell content (Table 2; selected ion intensity maps from TMA shown in Figure S2). Two peptides (m/z values in Table S3) from the proteins, COL1A2, COL6A3, HSPA5, HIST1H2BC, KRT9, AHNAK and NID2, were present at significantly higher intensities in tumor cell-rich areas in high-risk neuroblastomas.

**Figure 3:**
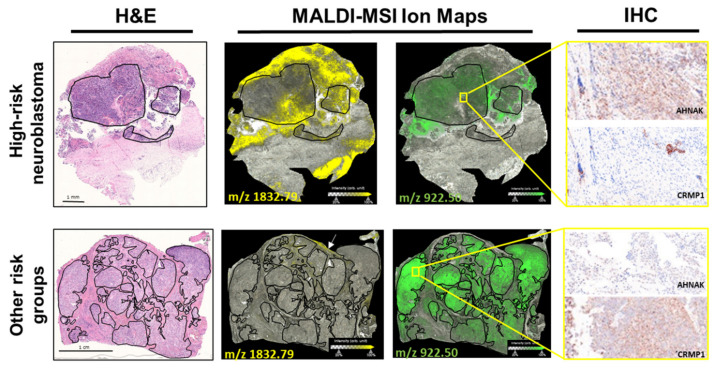
Validation of two discriminative protein markers for neuroblastoma risk in tissue sections. Shown are representative tissue sections from neuroblastoma designated high-risk (HR) and as other risk groups (nHR). MALDI-MSI ion maps for one peptide (m/z 1832.79 Da) assigned to AHNAK and one peptide (m/z 922.50 Da) assigned to CRMP1 are shown next to the corresponding sections stained with hematoxylin and eosin (H&E) for orientation. Black lines border areas with >80% tumor cell content. Immunohistochemical (IHC) detection of AHNAK and CRMP1 is shown for the regions surrounded by the yellow squares in the expanded image (400× magnification).

This group is enriched for extracellular matrix components (COL1A2, COL6A3 and NID2) and proteins associating with or regulating cytoskeletal proteins (AHNAK) as well as a cytoskeletal protein (KRT9). The two peptides assigned to CRMP1 had significantly lower intensities in tumor cell-rich areas from high-risk neuroblastomas compared to lower risk classifications. We selected two representative proteins from those identified for validation in adjacent neuroblastoma tissue sections using immunohistochemistry. AHNAK expression was higher in tumor cell-rich areas in high-risk neuroblastomas than in the lower risk groups (Figure 3). Reciprocally, CRMP1 expression was lower in high-risk neuroblastomas compared with lower risk groups (Figure 3), validating our MALDI-MSI profiling results. Our data strongly support that the 1832.79 m/z peak captured by MALDI-MSI have a higher intensity in tumor cell-rich regions of high-risk neuroblastomas is a tryptic peptide from AHNAK, an approximately 700 kD scaffold protein not previously published in the context of neuroblastoma. It was initially reported to be associated with neuroblast differentiation (reviewed in Davis2014) [41], but more recent studies have also pointed to an important role in promoting cellular proliferation, migration and epithelial-mesenchymal transition (EMT), processes leading to a short disease-free survival time and poor outcome of aggressive cancers including pancreatic ductal adenocarcinoma [42]. Likewise, the relatively low intensity 922.50 m/z peak in MALDI-MSI of high-risk neuroblastomas is a tryptic peptide from CRMP1, a marker for neuronal differentiation that is involved in neuronal outgrowth and guidance. It has been previously used in mRNA panels for neuroblastoma MRD and tumor-initiating cells [43,44,45,46]. These findings strongly support the correct identification of these 8 proteins as sources for 18 tryptic peptides detected by MALDI-MSI in FFPE neuroblastoma tissue sections and validate AHNAK and CRMP1 as discriminatory protein markers with potentially interesting and plausible biological roles.

Taken together, MALDI-MSI is feasible for the investigation of molecular cell phenotypes in histologically homogeneous appearing areas of high-risk neuroblastoma. Our data show these cells to be molecularly heterogeneous, and we identified discriminatory peptide signatures for high-risk neuroblastoma. From the discriminatory peptides, 18 could be assigned to 8 proteins, and differential AHNAK and CRMP1 expression was immunohistochemically validated in tissue sections. AHNAK shows intense and distinct staining in the tumor cell-rich regions in high-risk neuroblastomas in comparison to other risk groups (slight staining). In contrast, CRMP1 staining is intense in tumor cell-rich regions of neuroblastomas with other risk designations and only exhibited slight staining in the high-risk group. A detailed analysis of their biological roles in neuroblastoma is warranted.

## 3. Discussion

MALDI-MSI is a unique mass spectrometric technique that combines spatial molecular analysis with conventional histological assessment. Neither labels nor prior knowledge of molecular targets is necessary to simultaneously analyze the distribution of hundreds of peptides within a tissue, and sample preparation is automated and relatively simple. These advantages make MALDI-MSI an optimal tool to identify biomarkers and explore tumor complexity. We have previously used MALDI-MSI on epithelial ovarian cancer samples to discriminate among four different histotypes [48] and identify a proteomic signature in early-stage disease that is a prognostic marker for recurrence [49]. Here, we applied this technique to expose spatially resolved proteomic changes directly on intact neuroblastoma FFPE tissue sections. The acquired spatial peptide signatures resulted in 11 identified proteins, most of which are associated with the extracellular matrix and cytoskeleton, which enabled us to distinguish high-risk neuroblastomas from the tissue sections independently of conventional histology. Differential expression of the identified discriminative proteins, AHNAK and CRMP1, was immunohistochemically confirmed in sections, and discriminative spatial intensities of m/z peaks were validated in microarrayed tissue cores from tumor cell-rich regions in neuroblastomas. Importantly, we show that MALDI-MSI is capable of detecting molecular heterogeneity on the protein level in neuroblastoma tissue sections.

Due to their heterogeneous distribution throughout the whole tissue sections, not all peptides detected by MALDI-MSI in the whole sections were detected in cores in the tissue microarray (Figure S3). Depending on the area of the entire tumor from which the core is obtained, this information can be lost, pointing to significant limitations in the use of tissue microarrays to detect tumor heterogeneity in comparison to MALDI-MSI on whole tissue sections as a new, more comprehensive and precise diagnostic option. Several studies demonstrate that MALDI-MSI in a powerful tool to aid pathology for different cancer types [26,50,51]. Our study emphasizes that the investigation of whole tumor tissue sections are promising to directly explore molecular tumor heterogeneity. Different areas in a tumor section, while being homogeneous in morphological structure, can contain differences in molecular composition [52,53]. Previous studies demonstrate that MALDI-MSI is suitable to determine molecular subtypes in high-grade serous ovarian cancer [31,49] or to perform tumor classification. MALDI-MSI is shown here to be suitable to acquire spatial peptide signatures with potential as tools to directly examine molecular heterogeneity from diagnostic neuroblastoma tissue sections and potentially assist discrimination of high- or ultrahigh-risk disease after testing in a larger patient cohort.

International risk classification of neuroblastoma, based on clinical criteria plus *MYCN* amplification and recently complemented by transcriptomic parameters, has proven its usefulness for making therapy decisions and for disease management. Adding diagnostic information on the protein level might have the potential to further improve fine-tuning and the precision of current risk classification approaches. With this paper, we provide the proof-of-concept for the technical feasibility of this approach. Even more important is the consideration of tumor heterogeneity for the future selection of reliable prognostic or predicative biomarkers and signatures.

Tumors are complex tissues interposing cancer cells with distinct cell types and structures including extracellular matrix, stromal cells, blood vessels and cellular immune components. Neighboring cells in the tumor stroma, best described by combining proteomic profiling with histological evaluation, also influence tumor actions and phenotypes [54]. This diversity of cellular and molecular composition results in intratumor heterogeneity as a key factor contributing to therapeutic failure, drug resistance and recurrence [55]. Neuroblastoma proteomes have been previously studied using tandem LC-MS in bulk tissue homogenates from each tumor sample, and have defined large-scale, up- or down-regulated proteins associated with high risk [56,57]. The most commonly used (LC-MS, 2-dimensional electrophoresis) proteomic methods use tissue homogenates and cannot assign protein alterations to morphological structures. Due to the high intratumor heterogeneity, information about protein alterations may be lost.

In addition to providing proof-of-concept for the technical feasibility of MALDI-MSI, the potential risk classification-relevant peptide signatures of neuroblastoma are described to open new avenues to assess tumor heterogeneity. Our data also identified two specific proteins with potentially important roles in neuroblastoma biology and disease course. Our data showed a lower intensity distribution of CRMP1 in high-risk neuroblastomas and reciprocally higher intensity distribution in low- and intermediate-risk neuroblastomas. This is well in line with the reported role of CRMP1 in neuronal differentiation and its previous use as a marker gene in neuroblatoma gene expression panels as well as its usefulness as a prognostic and diagnostic marker in other cancers [58]. A detailed functional assessment of the biological role of CRMP1 in neuroblastoma is warranted in subsequent studies, but is beyond the scope of this paper. The lower mass accuracy of the presented workflow makes it more susceptible to false-positive protein assignments. Consequently, selected m/z values were matched to their source proteins to examine whether the differential peptide signature includes peptides from biologically feasible proteins in neuroblastoma, and subsequently validated their differential expression in tumor sections using immunohistochemistry. High- or ultrahigh-resolution mass spectrometry combined with microproteomics from microdissected regions in consecutive tissue sections is a promising technology for accurate extensive spatial proteomic characterization and quantification [28,29]. However, its use in high-throughput workflows, such as for large sample cohorts, is limited. This is an important prerequisite to explore potential clinical applications for alternative or improved risk assessment in a large tumor sample cohort.

We identified AHNAK as a marker protein highly expressed in high-risk neuroblastoma, from which tryptic peptides have high intensity distributions in tumor cell-rich regions of sections analyzed by MALDI-MSI. AHNAK has not been previously associated with neuroblastoma, but has been implicated in several cellular functions associated with cancer, including being listed one of six putative cancer genes involved in the evolution of nine cancer types across 3000 cancer genomes [59]. Most interestingly, AHNAK has been reported to be associated with enhanced proliferation and migration in rhabdomyosarcoma [60] among other cancers as well as supporting EMT in hepatoblastoma [61], endometrial [62] and lung [63] cancer cells as well as pancreatic ductal adenocarcinoma [42] and gastric cancer [64]. A similar role in neuroblastoma would be well in line with our previous observations that several signaling elements involved in EMT regulation are mutated in relapsed neuroblastomas [13]. However, the role of AHNAK in cancer appears to be tissue-specific, as other reports also point to a potential role as a tumor suppressor in glioma [65] and breast cancer [66]. This may be due to the fact that AHNAK achieves its breadth of activity by being a large protein that moderates multiprotein complex function by acting as a scaffold to tether activity either in the nucleus or at the plasma membrane and having its own phosphorylation sites that alters interactivity and intracellular localization [41]. The neuroblastoma-specific biological role of AHNAK has to be evaluated in subsequent detailed studies beyond the scope of this paper. Interestingly, AHNAK peptide intensity in MALDI imaging of low- and intermediate-risk neuroblastoma sections was also occasionally high in areas with <80% tumor cell content. While we can only speculate about the source of expression, these could represent subclones of molecularly evolving neuroblastoma cells or groups of neuroblastoma cells that are held back from evolving by influences of the surrounding stroma.

AHNAK was also occasionally upregulated in some area of the tumor stroma. Due to the barrier of natural structure including connective tissues, fibroblasts, immune cells and vasculature, common mass spectrometry methods are limited and cannot expose the molecular composition of the stromal compartment. MALDI-MSI is able to map protein changes in both areas that clearly exhibit the cellular interaction between malignant cancer cells and their environment and provides new insights for understanding neuroblastoma tumorigenesis and progression.

## 4. Materials and Methods

### 4.1. Patient and Sample Cohort

All samples were collected from primary neuroblastomas (located in the adrenal) for diagnostic purposes and were conserved in the local pathology departments as FFPE tissue blocks. Diagnosis of neuroblastoma was confirmed by an experienced reference pathologist and risk classification for patients, performed by the national neuroblastoma trial group, was based on definitions of the German BFM-NB2004 Trial and recommendations by the German Society for Pediatric Oncology and Hematology (GPOH). The comprehensive patient data set included sex, age, tumor INSS stage at diagnosis, presence or absence of *MYCN* amplification in the diagnostic tumor sample (detected by FISH), INRG risk classification and outcome, in particular diagnosis of relapse and death of disease (Table 3). Follow-up time for patients in this cohort was at least 4 years or until death of disease. Tissue areas with >80% tumor cell content were identified by the pathologist for both stancing tissue cores to create the tissue microarray and annotating sections analyzed by MALDI-MSI. Sample numbers 1–5 (high-risk) and 10–13 (other risk designations, Table 3) were used in an analyses of whole tissue sections (MALDI-MSI and immunohistochemistry). Cores from sample numbers 4, 10, 12 and 13 were also stanced for the tissue microarray together with tissue cores from 6 tumor samples from independent patients. Tumor cores were removed from FFPE tissue blocks using a 1.0-mm diameter hollow needle as tissue cores, which were arrayed in a recipient paraffin block (Table 3).

### 4.2. Tissue Immunohistochemistry

FFPE tissue sections (whole sections) were dewaxed and subjected to a heat-induced epitope retrieval step. Endogenous peroxidase was blocked by hydrogen peroxide prior to incubation with a monoclonal antibody against human CRMP1 (EP14521, Abcam, Cambridge, UK), followed by incubation with EnVision+ HRP-labeled polymer (Agilent Technologies Inc., Santa Clara, CA, USA) and visualization using the OPAL system (Akoya Biosciences Inc., Marlborough, MA, USA) according to manufacturer’s instructions. After protein inactivation, sections were incubated with a polyclonal antibody against human AHNAK (PA5-53890, Invitrogen, Thermo Fisher Scientific, Waltham, MA, USA), followed by incubation with the EnVision+ polymer (Agilent Technologies Inc.) and visualization using the OPAL system. Nuclei were stained with 4′,6-diamidine-2′-phenylindole dihydrochloride (DAPI; Merck KGaA, Darmstadt, Germany) and slides were mounted in Fluoromount G (Southern Biotech, Birmingham, AL, USA). Multispectral images were acquired using a Vectra^®^ 3 imaging system (Akoya Biosciences Inc., Malborough, MA, USA).

### 4.3. MALDI-MSI

All FFPE tissue sections (whole sections and tissue microarrays) were cut to 6-µm thickness by microtome (HM325, Thermo Fisher Scientific, Waltham, MA, USA.) and mounted onto conductive glass slides coated in indium tin oxide (Bruker Daltonik GmbH, Bremen, Germany). Sections were preheated to 80 °C for 15 min before deparaffinization. Paraffin was removed in xylene, and tissue sections were processed through 100% isopropanol and successive hydration steps of 100% ethanol followed by 96%, 70%, and 50% ethanol, each for 5 min. Sections were fully rehydrated in Milli-Q-purified water (Merck KGaA, Darmstadt, Germany). Heat-induced antigen retrieval was performed in MilliQ-water for 20 min in a steamer. After drying slides for 10 min, tryptic digestion was performed. An automated spraying device (HTX TM-Sprayer, HTX Technologies LLC, ERC GmbH, Riemerling, Germany) was used to deliver, onto each section, 16 layers of tryptic solution (20 µg Promega® Sequencing Grade Modified Porcine Trypsin in 800 µL digestion buffer-20 mM ammonium bicarbonate with 0.01% glycerol) at 30 °C. Tissue sections were incubated for 2 h at 50 °C in a humidity chamber saturated with potassium sulfate solution, then the HTX TM Sprayer applied 4 layers of the matrix solution (7 g/L a-cyano-4-hydroxycinnamic acid in 70% acetonitrile and 1% trifluoroacetic acid) at 75 °C. MALDI imaging was conducted on the rapifleX^®^ MALDI Tissuetyper^®^ (Bruker Daltonik GmbH, Bremen, Germany) in reflector mode with the detection range of 800–3200 m/z, 500 laser shots per spot, a 1.25 GS/s sampling rate and raster width of 50 μm. FlexImaging 5.1 and flexControl 3.0 software (Bruker Daltonik GmbH) coordinated the MALDI imaging run. External calibration was performed using a peptide calibration standard (Bruker Daltonik GmbH). The matrix was removed from tissue sections with 70% ethanol after MALDI imaging, and sections were stained with hematoxylin and eosin for histology. Tumor regions with >80% tumor cells were digitally annotated by a pathologist in the SCiLS cloud and transferred into SCiLS Lab software (Version 2019c Pro, Bruker Daltonik GmbH).

### 4.4. Protein Identification by Electrospray Ionization Tandem Mass Spectrometry

Protein identification for m/z values was performed on adjacent tissue (tumor cell-rich regions) sections using a bottom-up nano-liquid chromatography electrospray ionization tandem mass spectrometry approach as previously described [67]. Similar to their preparation for MALDI-MSI, sections were preheated to 80 °C for 15 min before deparaffinization. Paraffin removal, antigen retrieval and tryptic digest were carried out as for MALDI-MSI. After incubation at 50 °C in a humidity chamber saturated with potassium sulfate solution for 2 h, peptides were extracted from tumor cell-rich regions separately from each tissue section into 40 μL of 0.1% trifluoroacetic acid and incubated for 15 min at room temperature. Digests were filtered using a ZipTip^®^ C18 following the manufacturer’s instructions, and the eluates were vacuum concentrated (Eppendorf^®^ Concentrator 5301, Eppendorf AG, Hamburg, Germany) and reconstituted separately in 20 µL 0.1% trifluoroacetic acid, from which 2 µL were injected into a NanoHPLC (Dionex UltiMate 3000, Thermo Fisher Scientific) coupled to an ESI-QTOF ultrahigh-resolution mass spectrometer (Impact II™, Bruker Daltonic GmbH, Bremen, Germany). The peptide mixture was loaded onto an Acclaim PepMap™ 100 C18 trap column (100 µm × 2 cm, PN 164564, Thermo Fisher Scientific) and calibrated with 10 mM sodium hypofluorite (flowrate 20 µL/h) before separation in an Acclaim PepMap™ RSLC C18 column (75 µm × 50 cm, PN 164942, Thermo Fisher Scientific) with an increasing acetonitrile gradient 2–35% in 0.1% formic acid (400 nL/min flow rate, 10–800 bar pressure range) for 90 min while the column was kept at 60 °C. Released charged peptides were detected by a tandem mass spectrometer using a full-mass scan (150–2200 m/z) at a resolution of 50,000 FWHM. AutoMS/MS InsantExpertise was used to select peaks for fragmentation by collision-induced dissociation. Acquired raw MS/MS spectra were converted into mascot generic files (.mgf) for amino acid sequences using ProteoWizard software [68] and were used to search the human Swiss-Prot database using the Mascot search engine (version 2.4, MatrixScience Inc. Boston, MA, USA) with the significance threshold of *p* < 0.05 and the settings for trypsin as the proteolytic enzyme; a maximum of 1 missed cleavage; 10 ppm peptide tolerance; peptide charges of 2+, 3+ or 4+; oxidation allowed as variable modification; 0.8 Da MS/MS tolerance and a MOWSE score >13 to identify the corresponding protein. MOWSE (for MOlecular Weight SEarch) is a method for identifying proteins from the molecular weight of peptides created by proteolytic digestion and measured with mass spectrometry [47]. The probability-based MOWSE score formed the basis to develop Mascot, a proprietary software for protein identification from mass spectrometry data Mascot results were exported as.csv files (Table S4). To match aligned m/z values from MALDI-MSI (Table S1) with the peptides identified by nanoLC-MS/MS (Table S4), we developed an excel macro in-house (File S1). The macro was applied with settings accommodating previously described parameters [40]. Briefly, the comparison of MALDI-MSI and LC−MS/MS m/z values required the identification of >1 peptide (search mass window < 0.3 Da). Only peptides with the smallest mass differences in the mass window and a correlation ratio ≥0.30 were counted as a match. The peptides with highest MOWSE peptide scores and the smallest mass differences between MALDI-MSI and LC-MS/MS data were accepted as correctly identified.

### 4.5. MALDI-MSI Data Processing for Statistical Analyses

MALDI-MSI raw data were imported into the SCiLS Lab software version 2019c Pro (Bruker Daltonik GmbH) using settings preserving the total ion count and without baseline removal and converted into the SCiLS base data .sbd file and .slx file. An attribute table was built for sample number, tumor cell-rich regions, tumor INSS stage, *MYCN* amplification status in diagnostic tumor sample, or whether the molecular risk designation was high or other, and on patient age, sex and whether the patient experienced disease recurrence. Attributes were used to divide a dataset into independent datasets from different spatial spectral regions in tissue sections, or samples with different tumor or patient characteristics for analysis. Peak finding and alignment were conducted across a dataset (interval width = 0.3 Da) using a standard segmentation pipeline (SciLS Lab software) in maximal interval processing mode with TIC normalization, medium noise reduction and no smoothing (Sigma: 0.75) [69,70]. 

### 4.6. Statistical Analyses

The top-down segmentation using bisecting k-means clustering analysis was performed on the partitioned datasets from tissue sections or from only the regions with >80% tumor cells, as previously described [71], to defined peptide signatures. Both analyses used settings for 0.3 Da interval width, including all individual spectra, medium noise reduction and correlation distance. Discriminative MALDI-MSI m/z values from tumor cell-rich regions were identified using supervised ROC analysis on the partitioned datasets from tissue regions with >80% tumor cells. Area under the ROC curve (AUC) varies between 0 and 1, where values close to 0 and 1 indicates peptides to be discriminatory and 0.5 indicates no discriminatory value. Since the number of m/z values from the groups to be compared must be similar for this analysis, 35,000 m/z values were randomly selected per group. For those peptides with an AUC >0.7 or <0.3, a univariate hypothesis test (Wilcoxon rank sum test) was used to test the statistical significance of m/z values. Peptides with *p*-values < 0.001 and a peak correlation ratio ≥0.30 were selected as candidate markers. Supervised principal component analysis (PCA) was conducted to define characteristic peptide signatures differentiating between tumor regions with >80% tumor cell content from high or other risk groups. The data were scaled for PCA in a level scaling model. Only m/z values with AUC >0.8 or <0.2 and *p* < 0.001 were used as peak intervals for PCA using settings to create five components and use settings to use an interval width of ±0.3 Da, maximal interval processing mode, normalization to total ion count, and no noise reduction. ROC analysis was also used in validation experiments to identify discriminative m/z values (defined in data sets from whole sections) using MALDI-MSI data (2500 m/z values randomly selected per group) from arrayed tumor cores. The Wilcoxon rank sum test was used to test the statistical significance of m/z values. Peptides with significant differences (*p*-value < 0.001) in the Wilcoxon test with a peak correlation ratio ≥0.30 were selected as candidate markers (significant correlations *p* < 0.05; Pearson’s correlation analysis [72]. All Figures were created using the SCiLS Lab software (Bruker, Bremen, Germany).

## 5. Conclusions

Molecular intratumor heterogeneity in high-risk neuroblastoma most likely contributes to therapy response and the clinical disease course, and is a challenge for risk assessment at initial tumor diagnosis. This pilot study demonstrates that (1) MALDI-MSI can visualize molecular tumor characteristics on the protein level associated with current risk classification directly in FFPE tumor tissue sections; (2) MALDI-MSI was able to explore spatial proteomic changes and directly identify molecular tumor heterogeneity in tumor sections; and (3) combined with nanoLC-MS/MS, this approach can identify differentially expressed new protein biomarkers in high-risk neuroblastomas (versus lower risk groups), which might have an important role in neuroblastoma biology and/or progression. We provide proof-of-concept for the usefulness of this innovative technology in assisting risk classification and assessment of tumor heterogeneity on the protein level, as well as identification of new biomarkers with potential relevance for an increased understanding of neuroblastoma biology.

## Figures and Tables

**Table 1:** Treatment classification of neuroblastoma patients.

INSS/INRG Staging	Age at Diagnosis (Months)	*MYCN* Status	Chromosome 1p Status	Treatment Risk Group
1		not amplified	normal	Low
		amplified		High
2		not amplified	normal	Low
			deletion/imbalance	intermediate
		amplified		High
3	<24	not amplified	normal	Low
	≥24	not amplified	normal	intermediate
		not amplified	deletion/imbalance	
		amplified		High
4s/MS	<18	not amplified	normal	Low
		amplified		High
4/M	<18	not amplified		intermediate
	≥18	amplified		High

INSS = International Neuroblastoma Staging System. INRG = International Neuroblastoma Risk Group (INRG) Staging System.

**Table 2:** Differential intensity distributions of peptides (MALDI-MSI) and their corresponding proteins in tissue sections from neuroblastomas in high or other risk groups.

MALDI IMS m/z Value	ROC [AUC] for High Versus other Risk *	ROC [AUC] HR/nHR TMA ^†^	Significance Rating-WRS	LC-MS/MS [Mr + H^+^ cal.]	Scores ^§^	Deviation [Da]	Correlation Coefficient	Protein Symbol	Protein
868.4930	0.85	0.73	<0.001	868.46	48.1	0.03	0.38	COL1A2	Collagen type I alpha 2 chain
1562.7700	0.91	0.74	<0.001	1562.79	127.	0.02	0.64		
2026.9100	0.86	0.73	<0.001	2027.02	65.8	0.11	0.36		
1459.8500	0.72	0.66	<0.001	1459.86	40.5	0.01	0.38	COL6A3	Collagen type VI alpha 3 chain
2056.9200	0.88	0.63	<0.001	2057.04	59.4	0.12	0.32		
766.4820	0.08	0.28	<0.001	766.46	21.7	0.03	0.44	CRMP1	Collapsin response mediator protein 1
922.4990	0.14	0.34	<0.001	922.51	22.3	0.02	0.40		
1833.9900	0.87	0.67	<0.001	1833.91	65.1	0.08	0.40	HSPA5	Heat shock protein family A (Hsp70) member 5
2042.2200	0.85	0.73	<0.001	2042.05	25.6	0.17	0.32		
1477.8600	0.90	0.75	<0.001	1477.79	28.1	0.07	0.41	HIST1H2BC	H2B clustered histone 4
1743.6800	0.82	0.58	<0.001	1743.82	96.2	0.14	0.58		
1775.7900	0.90	0.70	<0.001	1775.81	123.	0.02	0.55		
1586.7700	0.90	0.74	<0.001	1586.77	89.4	0.00	0.47	KRT9	Keratin 9
2705.2800	0.86	0.78	<0.001	2705.16	67.9	0.12	0.44		
1267.5000	0.87	0.74	<0.001	1267.65	63.9	0.12	0.38	AHNAK	AHNAK nucleoprotein
1832.7900	0.92	0.70	<0.001	1832.88	44.7	0.09	0.39		
1706.7800	0.87	0.74	<0.001	1706.78	31.2	0.00	0.31	NID2	Nidogen 2
2455.3600	0.79	0.72	<0.001	2455.17	34.9	0.19	0.33		

* Calculated from data obtained from regions in whole tissue sections with >80% tumor cell content. ^†^ TMA = tissue microarray (arrayed neuroblastom tissue cores from areas with >80% tumor cell content). ^§^ MOlecular Weight Search score [47].

**Table 3:** Clinicopathological characteristics for our patient cohort.

ID	Sex	Age (Years)	INSS Stage	*MYCN* Amplification	Risk Classification (at Diagnosis)	Disease Recurrence	Death	Metastasis
1	F	0.3	3	+	high	-	-	No
2	M	0.6	2	+	high	-	-	No
3	M	1	3	+	high	-	+	No ^†^
4	M	1.4	4	+	high	-	-	Yes
5	F	1.2	4	+	high	+	+	Yes
6	M	2.8	4	+	high	+	+	Yes
7	M	7.8	4	-	high	-	-	Yes
8	F	8	4	+	high	-	-	Yes
9 ^‡^	M	1.2	3	-	high	+	-	No ^‡^
10	F	2.4	1	-	low	-	-	No
11	F	0.8	4	-	intermediate	-	-	Yes
12	M	0.1	4s	-	low	-	-	Yes
13	F	0.1	3	mosaic	low	-	-	No
14	F	5.9	3	-	intermediate	-	-	No
15	M	1.9	2	-	low	-	-	No

^†^ Disease in this patient later metastasized and was upgraded to INSS stage 4. ^‡^ This patient had multiple relapses after first-line therapy and was treated for high-risk disease in relapse therapy.

## Data Availability

Data are contained within the article or supplementary material. The MALDI-MSI data presented in this study are available on request from the corresponding author.

## Supplementary Materials

The following are available online at https://www.mdpi.com/article/10.3390/cancers13133184/s1 are Figure S1: Representative average spectra of whole sections and malignant regions of HR and non-HR neuroblastoma tissue sections. Figure S2: Discriminatory peptide signatures within tumor cell-rich regions in high or other risk groups of neuroblastoma. Figure S3: Ion maps of m/z values for CRMP1 and AHNAK in whole neuroblastoma sections and their validation in selected cores from the tissue microarray. Table S1: Aligned m/z values from cell-rich tumor region in neuroblastoma sections and tumor microarray cores. Table S2: Segmentation map of the tumor cell-rich regions in sections from high-risk neuroblastoma (HR) and neuroblastomas from other risk groups (nHR). Table S3: Identified discriminative MALDI-MSI m/z values by using nanoLC-MS/MS. Table S4: Protein identification by LC-MS of neuroblastoma whole tissue section. File S1: Excel macro codes for linking MALDI-MSI data and LC-MS/MS data.
