# Supplementary material for: Discovery of Spatial Peptide Signatures for Neuroblastoma Risk Assessment by MALDI Mass Spectrometry Imaging
Source: Cancers (Basel). 2021 Jun 25;13(13):3184. doi: 10.3390/cancers13133184 (PMC8269054; doi:10.3390/cancers13133184)
Supplement: Supplementary file 1 [file cancers-13-03184-s001.zip › cancers-1214542 supplementary final/Supplementary_Figures.pdf]

Supplementary Information

Discovery of Spatial Proteomic Signatures for Neuroblastoma Risk Assessment by MADLI Imaging Mass Spectrometry

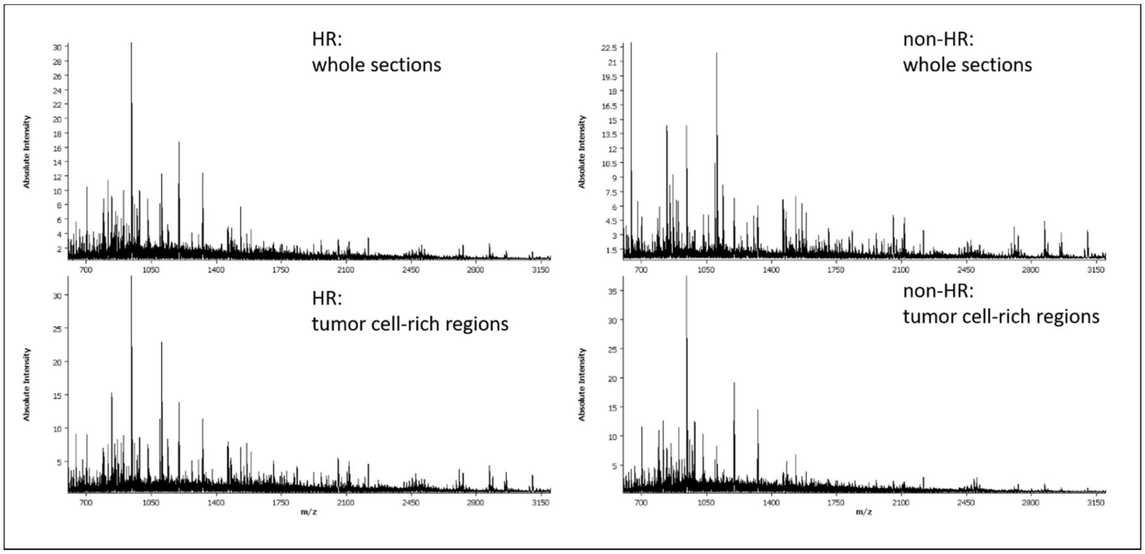

Figure S1: Representative average spectra of whole sections and tumor cell-rich regions of HR and non-HR Neuroblastoma tissue sections**.**

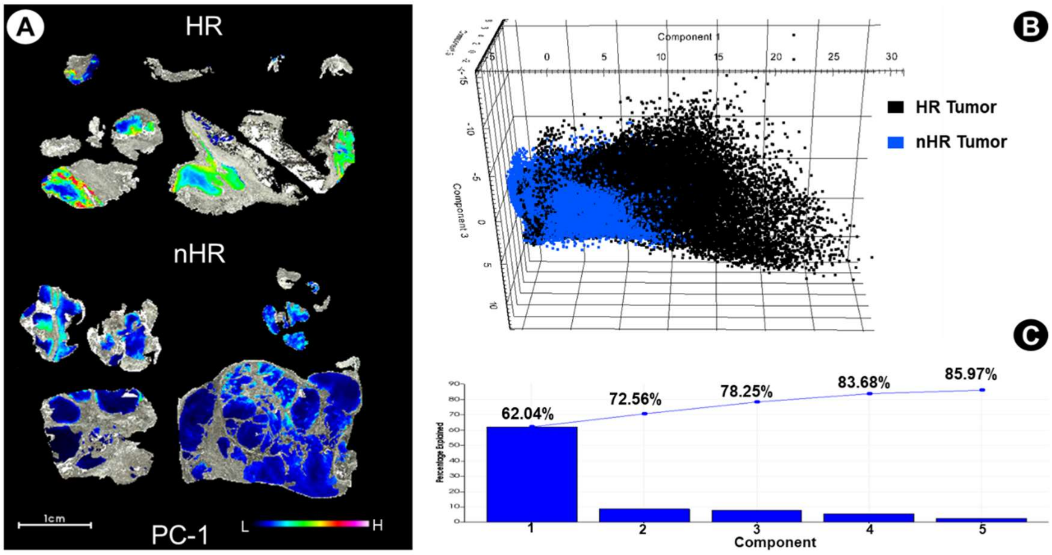

Figure S2. Discriminatory proteomics signatures within tumor cell-rich regions in high or other risk groups of neuroblastoma. **A.** Intensity of the first principal component (PC1) is shown in the ion intensity maps are shown for sections from high-risk (HR) neuroblastomas and neuroblastoma from lower risk groups (nHR). **B.** The corresponding 2D scores plot of MALDI IMS derived spectra (m/z values AUC >0.7, <0.3) for the components 1, 2 and 3 shows possibility of distinguishing proteomic signature for HR (black cluster) and nHR (blue cluster) in tumor cell-rich regions. **C.** PCA variance plot shows that 62% of variance explained by PC-1.

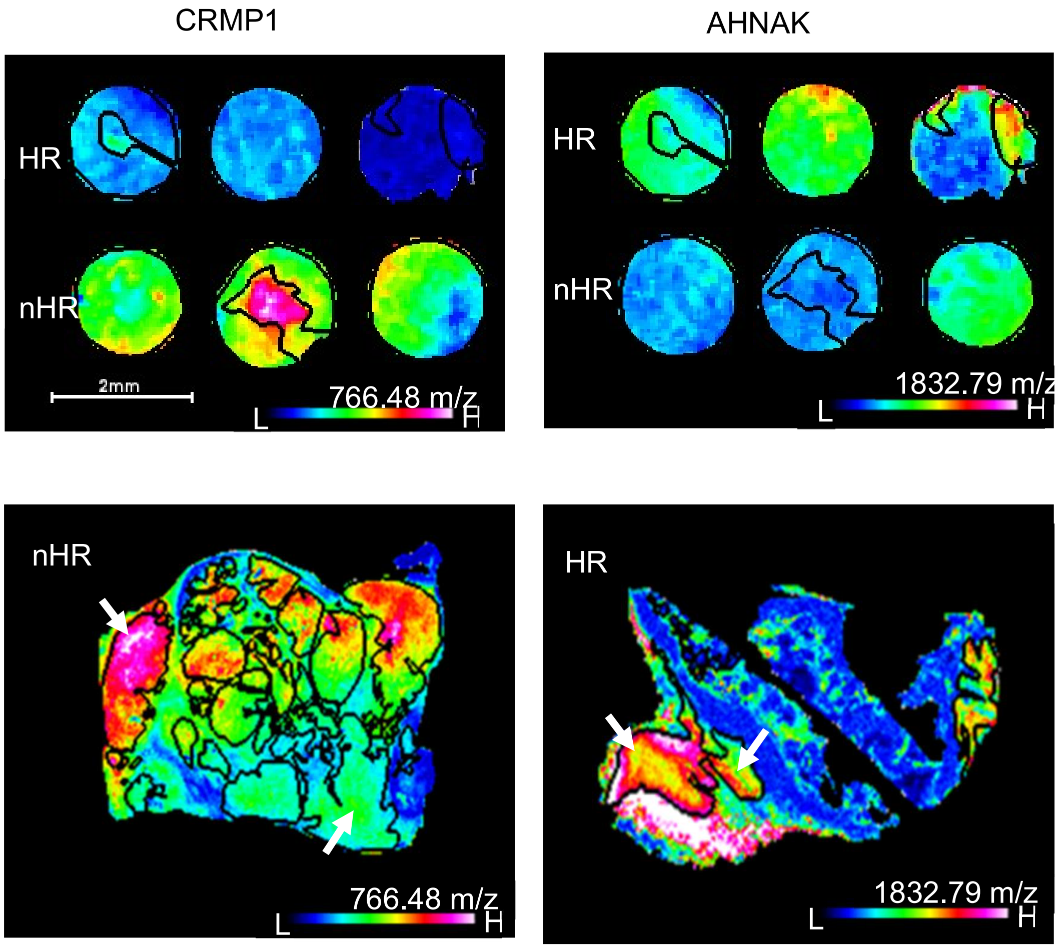

Figure S3: Ion maps of m/z values for CRMP1 and AHNAK in whole neuroblastoma sections and their validation in selected cores from the tissue microarray. Intensity distributions of one peptide from CRMP1 (m/z value 766. 48 Da) and one peptide from AHNAK (m/z value 1832.79 Da) are shown in selected tumor cores from the neuroblastoma tissue microarray (upper images) and whole tissue sections (lower images) from neuroblastomas designated high-risk (HR) or in other risk groups (nHR). White arrows point out areas of heterogenic ion distribution in the whole tumor sections.
