# Supplementary material for: Discovery of Spatial Peptide Signatures for Neuroblastoma Risk Assessment by MALDI Mass Spectrometry Imaging
Source: Cancers (Basel). 2021 Jun 25;13(13):3184. doi: 10.3390/cancers13133184 (PMC8269054; doi:10.3390/cancers13133184)
Supplement: Supplementary file 1 [file cancers-13-03184-s001.zip › cancers-1214542 supplementary final/Supplementary_File S1.pdf]

Software: excel as the vision of 2016

Funktion: this macro links the m/z values of MALDI-IMS with m/z values of LC-MS/MS and present identified peptides for MALDI-IMS data.

```
Sub MH()
    Application.ScreenUpdating = False
    Application.AskToUpdateLinks = False
    Application.EnableEvents = False
    Application.DisplayAlerts = False
    Dim crr
    Dim drr
    Dim dic(1 To 3)
    For i = 1 To UBound(dic)
        Set dic(i) = CreateObject("scripting.dictionary")
    Next
    With ThisWorkbook.Worksheets("MH")
        lr = .Range("A" & .Rows.Count).End(xlUp).Row
        lc = .Cells(1, .Columns.Count).End(xlToLeft).Column
        .[a1].Resize(lr, 1).Value = .[a1].Resize(lr, 1).Value
        .[a1].Resize(lr, lc).Sort key1:=.[a1], order1:=1, Header:=xlYes
        arr = .[a1].Resize(lr, lc)
        For i = 2 To lr
            arr(i, 2) = Val(Split(arr(i, 2), " ")(0))
            temp_k = "A" & (arr(i, 1) * 10 ^ 12)
            temp_v = .Cells(i, 1).Resize(1, 7)
            If i = 52 Then
                Debug.Print i
            End If
            If dic(1).exists(temp_k) = False Then
                dic(1)(temp_k) = arr(i, 2)


                dic(2)(temp_k) = temp_v
                dic(3)(temp_k) = 1
            Else
                If dic(1)(temp_k) < arr(i, 2) Then
                    dic(1)(temp_k) = arr(i, 2)


                    dic(2)(temp_k) = temp_v
                    dic(3)(temp_k) = 1
                ElseIf dic(1)(temp_k) = arr(i, 2) Then
                    dic(3)(temp_k) = dic(3)(temp_k) + 1
                    ReDim drr(1 To dic(3)(temp_k), 1 To 7)
                    For m = 1 To 7
                        drr(UBound(drr), m) = temp_v(1, m)
                    Next
                    temp_v = dic(2)(temp_k)
                    For m = 1 To UBound(drr) - 1
                        For n = 1 To UBound(drr, 2)
                            drr(m, n) = temp_v(m, n)
                        Next
                    Next
                    dic(2)(temp_k) = drr
                    Erase drr
                End If
            End If
        Next
    End With
    'Zhiyang Wu'
```

```
    With ThisWorkbook.Worksheets("MZ")
        lr = .Range("A" & .Rows.Count).End(xlUp).Row
        lc = .Cells(1, .Columns.Count).End(xlToLeft).Column
        brr = .[a1].Resize(lr, lc + 1)
        brr(1, lc + 1) = "FZ"
        For i = 2 To lr
            brr(i, lc + 1) = Split(brr(i, 1), " ")(0)
        Next
        .[a1].Resize(lr, lc).NumberFormatLocal = "@"
        .[a1].Resize(lr, lc + 1) = brr
        .[a1].Resize(lr, lc + 1).Sort key1:=.Cells(1, lc + 1), order1:=1, Header:=xlYes
        .Range("A:A").Offset(, lc).Clear
        brr = .[a1].Resize(lr, lc)
        For i = 2 To lr
            brr(i, 1) = Val(Split(brr(i, 1), " ")(0))
        Next
    End With
    ReDim crr(1 To UBound(brr) * 100, 1 To UBound(arr, 2) + UBound(brr, 2))
    k2 = dic(2).keys
    For i = 0 To UBound(k2)
        k2(i) = --Replace(k2(i), "A", "")
    Next
    t = 1
    For i = 1 To UBound(brr, 2)
        crr(t, i) = brr(1, i)
    Next
    For i = UBound(brr, 2) + 1 To UBound(crr, 2)
        crr(t, i) = arr(1, i - UBound(brr, 2))
    Next
    For j = 1 To dic(2).Count
        If Abs(arr(2, 1) - k2(j - 1) / 10 ^ 12) < 1 Then
            flag = j
            Exit For
        End If
    Next
    'zhiyang.wu@charite.de'
    For i = 2 To UBound(brr)
        t1 = t
        For j = flag To 1 Step -1
            If brr(i, 1) - k2(j - 1) / 10 ^ 12 < 1 Then
                If Abs(brr(i, 1) - k2(j - 1) / 10 ^ 12) < 1 Then
                    For m = 1 To dic(3)("A" & k2(j - 1))
                        t = t + 1
                        For l = 1 To UBound(brr, 2)
                            crr(t, l) = brr(i, l)
                        Next
                        temp_v = dic(2)("A" & k2(j - 1))
                        For l = UBound(brr, 2) + 1 To UBound(crr, 2)
                            crr(t, l) = temp_v(m, l - UBound(brr, 2))
                        Next
                    Next
                End If
            Else

                Exit For
            End If
        Next
```

```
        For j = flag + 1 To dic(2).Count
            If k2(j - 1) / 10 ^ 12 - brr(i, 1) < 1 Then
                If Abs(brr(i, 1) - k2(j - 1) / 10 ^ 12) < 1
Then
                    t = t + 1
                    For l = 1 To UBound(brr, 2)
                        crr(t, l) = brr(i, l)
                    Next
                    temp_v = dic(2)("A" & k2(j - 1))
                    For l = UBound(brr, 2) + 1 To
UBound(crr, 2)
                        crr(t, l) = temp_v(1, l - UBound(brr,
2))
                    Next
                End If
            Else
                flag = j
                Exit For

            End If
        Next
        If t1 = t Then
            t = t + 1
            For l = 1 To UBound(brr, 2)
                crr(t, l) = brr(i, l)
            Next
        End If

    Next
    With ThisWorkbook.Worksheets("RS")
        .UsedRange.Clear
        .[a1].Resize(t, UBound(crr, 2)) = crr
    End With
    MsgBox "Done"
    Application.ScreenUpdating = True
    Application.AskToUpdateLinks = True
    Application.EnableEvents = True
    Application.DisplayAlerts = True 'Zhiyang
Wu, zhiyang.wu@charite.de'
End Sub



Sub test()
    Set dic = CreateObject("scripting.dictionary")
    With ThisWorkbook.Worksheets("MH")
        lr = .Range("A"
& .Rows.Count).End(xlUp).Row
        lc
= .Cells(1, .Columns.Count).End(xlToLeft).Colu
mn
        .[a1].Resize(lr, 1).Value = .[a1].Resize(lr,
1).Value
        .[a1].Resize(lr, lc).Sort key1:=.[a1], order1:=1,
Header:=xlYes
        arr = .[a1].Resize(lr, lc)
        For i = 2 To lr
            dic(arr(i, 1)) = dic(arr(i, 1)) + 1
            .Cells(i, lc + 1) = dic(arr(i, 1))
        Next

    End With



End Sub
```
